# 30 Years Retrospective Review of Tuberculosis Cases in a Tuberculosis Dispensary in Bursa/Nilufer, Turkey (1985–2014): Changes of Epidemics^*^

**DOI:** 10.4084/MJHID.2016.059

**Published:** 2016-11-01

**Authors:** Kayıhan Pala, Harika Gerçek, Tuncay Aydin Taş, Rukiye Çakir, Sedef Özgüç, Timur Yildiz

**Affiliations:** 1 Public Health Department, Uludag University Faculty of Medicine, Bursa, Turkey; 2 Nilufer Tuberculosis Control Association Dispensary, Bursa, Turkey

## Abstract

**Objective:**

The aim of this study is to describe the epidemiological and clinical aspects of patients who applied to the Bursa Nilufer Tuberculosis Dispensary by investigating the trends in epidemics over three decades.

**Method:**

In this retrospective observational study, the records of all tuberculosis cases (1630 patients) treated in the last 30 years (1985–2014) at the Bursa Nilufer Tuberculosis Dispensary were examined and statistically analyzed.

**Results:**

Males comprised 65.2% of the patients. The ages of the patients ranged from 1 to 87 years, and the mean age was 37.4 (95% CI: 36.6–38.2). Among the cases, 86.7% were new infections and 74.1% were pulmonary tuberculosis. In the last decade, the education level, the percentage of patients who had received a BCG vaccination, the proportion of women and active employees among them increased (p<0.05), while it decreased among men (p<0.05). Clinical symptoms accompanying TB such as weakness, anorexia, weight loss, and cough, decreased to a statistically significant degree (p<0.05). In the last decade, the mortality rate was 3.6% and increased compared with previous decades (p<0.05). Mortality was higher among patients who were elderly, male, did not have a BCG scar or had a chronic disease (p<0.05).

**Conclusion:**

This study adds information about the change of TB epidemics in Turkey in the last 30 years. Further studies are needed to determine the risk factors associated with tuberculosis mortality and to evaluate the effectiveness control programs of this disease.

## Introduction

Tuberculosis (TB) is a disease that primarily affects the lungs, but it can spread to extrapulmonary organs through lymphogenic and hematogenous routes.^1^ Approximately one-third of the world population has an asymptomatic and non-infectious latent infection. About 10% of these asymptomatic patients progress to active disease and approximately 45% of individuals with the active disease die if it is not treated.^1^,^2^

The World Medical Association emphasizes that poverty fuels the spread of tuberculosis by causing limited access to primary health care services, inducing malnutrition, and inadequate living conditions; therefore, tuberculosis should be considered as a disease of poverty and inequality.^3^

Despite notable progress in the past decade, tuberculosis is still a public health concern in most of the countries within the World Health Organization (WHO) European Region. Countries outside of the European Union (EU) and European Economic Area (EEA) still suffer from high rates of TB and multidrug-resistant TB, while EU/EEA countries have a significant number of TB cases among vulnerable population groups, such as people of foreign origin and prisoners. In 2014, an estimated 340 000 incident cases of TB (range 320 000–350 000) occurred in the WHO European Region, equivalent to 37 cases (35–38) per 100 000 population. This number represents about 3.6% of the total burden in the world. About 83% of incident TB cases in 2014 occurred in the 18 high-priority countries.^4^ Turkey is one of the 18 high-priority countries.

The first data regarding the epidemiological situation of tuberculosis in Turkey pertained to the year 1950. TB mortality, which was 204/100.000 in 1950, decreased to 8.8/100.000 in 1980 and 1.6/100.000 in 2000. While the tuberculosis incidence was 177/100.000 according to the values for 1960, it dropped to 24/100.000 in 2002.^5^ In Turkey, the estimated TB prevalence was 22/100.000, the incidence was 18/100.000, and the mortality was 0.61/100.000 for 2014.^6^

In Turkey, the TB control program began in 1918 under the guidance of Tuberculosis Control Associations, which were voluntary organizations, and have been maintained via vertical structuring within the Ministry of Health and provincial organizations.^7^ Public Health Law (No. 1593, 1930) designated tuberculosis as a notifiable disease and made its treatment free of charge. In Turkey, information regarding the patients comes primarily from the records of Tuberculosis Control Dispensaries (TCDs). Valuable information resources are also the general death records and the results of epidemiologic studies. Since 2007, the Department of Tuberculosis Control has collected information regarding patients registered at the TCDs and has published them as reports.^8^

TCDs are the health institutions that provide diagnosis, treatment, follow-up and control, patient notification, registration, archiving and statistics, immunizations, screening, drugs, training, health education activities, social welfare, coordination, and consultancy.^9^ The TCD follows the guidelines of “Stop TB Strategy” and the “International Standards for Tuberculosis Care” adopted by the WHO.^10^

Bursa Nilufer Tuberculosis Control Dispensary was established as the Bursa Tuberculosis Control Association in 1948 and was included among public interest associations in 1949.^11^ Since its service building moved from the city center to the Nilufer district in 2003, the Association has provided services as the Nilufer Tuberculosis Control Association Dispensary.^12^

The dispensary case series are relevant for determining the current situation of tuberculosis in Turkish.

In this study, we analyzed socio-demographic characteristics, the clinical findings, diagnosis and treatment processes and treatment results of the patients who applied to Bursa Nilüfer Tuberculosis Control Dispensary (NTCD) in a period of thirty years.

## Material and Method

Bursa is the fourth most populous city (1.8 million) in Turkey and is located in Northwestern Anatolia in Turkey. Nilufer is one of the three central districts of Bursa which was established on the western side of the city. Nilufer is the newest and most planned and organized district of Bursa; it is the most rapidly urbanizing area of the city, and its population is slight over 300.000. The Nilufer ranks the first place amongst the districts of Bursa significantly contributing to the economy of Turkey and Bursa. The first Organized Industrial Zone of Turkey has been established within the district of Nilufer in 1961. There are twenty-three Family Health Centre, one Community Health Center, one Tuberculosis Control Dispensary and two public hospitals in Nilufer.

This descriptive study was carried out between June 2014 and February 2015. The files of all cases (1662 people) receiving tuberculosis treatment in Nilufer Tuberculosis Control Dispensary (NTCD) were reviewed, comprising the treatments of the last 30 years (1985–2014). Thirty-two patients who had started treatment of tuberculosis were later diagnosed carriers of another disease and then excluded from this study. Therefore only the data of 1,630 patients were evaluated.

TB cases were diagnosed both in Dispensary and public hospitals. Pulmonary TB was diagnosed by X-ray, smear microscopy of sputum and sputum culture. The most commonly used media for the isolation of tuberculosis were solid egg-based (Löwenstein–Jensen, Ogawa) and agar-based (Middlebrook 7H10 or 7H11) media; manual liquid synthetic (Middlebrook 7H9) and automated liquid media (Bact/Alert 3D, MGIT 960, VersaTREC). Non-pulmonary TB was diagnosed only in hospitals. According to legislation in Turkey, patients diagnosed of TB in hospitals referred to the regional dispensary for receiving TB treatment. In 30 years there was no change in the definitive TB diagnosis (X-ray, smear microscopy of sputum and sputum culture) in Dispensary; but there is no information in defining TB diagnosis methods in the hospitals in the dispenser records.

In this study, were retrospectively examined the tuberculosis patient registers, tuberculosis patient monitoring vouchers, patient examination forms and computer records. The data obtained were included in a data collection form consisting of 48 questions. The patients’ socio-demographic information (age, gender, marital status, educational status, profession, work conditions, social security status), the presence of chronic disease, TB history, the history of contact with a tuberculosis patient, the presence of a Bacillus Calmette-Guérin (BCG) scar, symptoms accompanying the diagnosis, case description, reason for examination, TB locations and treatment result were examined in this form. The underlying concepts and definitions used in this study were based on the “T. R. Ministry of Health Tuberculosis Diagnosis and Treatment Guidelines 2011”.^10^ The occupations of working patients were classified according to the International Standard Classification of Occupations ISCO 08.

Drug resistance has been evaluated in patients since mid-1990’s. TB resistance was determined by drug susceptibility testing. In the laboratory, drug susceptibility testing of TB isolates was performed by the proportion methods (agar based MB 7H10/11, Löwenstein Jensen); automated fluid systems (MGIT 960, VersaTREK) and molecular methods (Real-time PCR, reverse hybridization). Multidrug-resistant TB is resistant to at least isoniazid and rifampin. Patients with the following characteristics were considered at risk of MDR-TB:

- Failure of retreatment regimens,
- Chronic TB cases,
- Exposure to a known MDR-TB case,
- Failure of first-line chemotherapy,
- Relapse and return after default without recent treatment failure, and
- History of using poor or unknown quality TB drugs.

TB treatment has been changing for the last 30 years period in the dispensary. In the first two decades nine-month treatment regimens were applied (in the first two months isoniazid, rifampin, pyrazinamide, and ethambutol or streptomycin and then for 7 months isoniazid and rifampin). In the last decade, a six-months standard TB treatment was applied. Standard TB treatment includes the use of 4 drugs: rifampin, pyrazinamide, isoniazid, and ethambutol given for two months, followed by a rifampin/isoniazid continuation phase for an additional four months.

The permission for the study was received from Uludağ University, Faculty of Medicine, Ethics Committee (dated June 10, 2014, and numbered 2014-12/3).

The research data were evaluated using the SPSS 18.0 software package. The descriptive statistics, chi-square test, chi-squared test for trend and Fisher’s exact test were employed in the data analysis. A p-value less than 0.05 was considered significant.

## Results

The total number of 1630 TB cases received tuberculosis treatment in NTCD from 1985 through 2014. When the patients were grouped into three periods of ten years (classified according to when the patient file was opened), 627 patients were diagnosed from 1985–1994, 490 from 1995–2004, and 513 from 2005–2014.

65.2% of the patients, were male, and the male/female ratio was 1.88. The patients’ ages ranged from 1 to 87 years; the mean age was 37.4 (95% G.A.: 36.6–38.2). The gender and age distribution of the cases are shown in Table 1 and socio-demographic characteristics of the cases according to sex and ten-year periods are shown in Table 2.

According to Dispensary records, 1572 cases were born in Turkey (96.4%), the rest of them were born in eleven different countries (Bulgaria, Greece, Yugoslavia, Turkmenistan, Georgia, Macedonia, Saudi Arabia, Uzbekistan, Ethiopia, Azerbaijan, Iraq) except Syria. There were no refugees from Syria receiving TB treatment in the NTCD.

The most frequently systemic symptom observed was coughing (72.1%). Night sweating was observed in 49.6%, sputum in 46.3%, hemoptysis in 14.9%, fever in 7.9%, and side pain in 3.1%. Allegations of malaise, anorexia, weight loss and coughing decreased in the last decade compared with the previous ten-year periods; however, complaints of back pain increased in the years (Table 3).

The main characteristics of TB cases related to diagnosis and treatment are shown in Table 3.

The relapse rate was 1.7% in the 0–14 year age group compared with 15.4% in the 40 – 64 year age group (p<0.001); it was 11.8% in pulmonary TB cases compared with 3.8% in extra pulmonary TB cases (p<0.001). The relapse rate, BCG scar presence, and contact history are shown according to gender, age group and involvement site in Table 4.

Domestic contact was determined for all children (0–14 year age group). Among all adult females, the non-contact was 77.1%, domestic contact was 21%, and the non-domestic contact was 1.9 %; among all adult males, the non-contact was 83.6, the domestic contact was 14.4 %, and the non-domestic contact was 2% in men (p=0.014).

The site involvement in relation to gender, age group, and treatment success is shown in Table 5.

Involvement of the pleura was found in 11.5% of the cases, lymph node involvement in 9.2%, bone involvement in 1.6% and genitourinary system involvement in 1.6%.

All of the 26 deaths occurred in patients aged 40 years and older. The mortality rate was significantly higher among elderly (over 65) people compared to 40–64 age groups (p<0.001), males (p=0.038), patients without a BCG scar (p=0.012), patients with multi-drug resistant TB (p=0.033) and those with chronic diseases (p<0.001).

When the BCG scar was analysed according to ten-year periods, the proportion of patients with a BCG scar of the 15–39 year age group decreased from 72.9% in the first ten years to 55.7% in the most recent decade, and the proportions among those aged 40–64 years and over 65 years increased from 19.8% to 34.1% and from 1.0% to 6.9%, respectively (p<0.001).

Twenty-four percent (1035) of the TB patients had at least one chronic disease (No information about chronic diseases were available for 595 patients). The presence of chronic diseases in TB cases have risen from 11,3% in the first decade to 26,6% in the second decade and 37,6% in last decade respectively (p<0.001). The accompanying chronic diseases were: diabetes mellitus in 31.3%; hypertension in 16.9%; chronic obstructive pulmonary disease (COPD), asthma and respiratory illness including chronic bronchitis in 14.1% and heart disease in 9.2%.

Culture and/or microscopic examinations could not be performed for 22.7% of the patients with pulmonary TB. In these cases, TB was diagnosed with clinical suspicion, X-rays, and ex juvantibus therapy. The proportion of pulmonary TB cases with positive bacteriology significantly increased (p<0.001) over the most recent decade (Table 6).

Among the new pulmonary TB cases with positive bacteriology, treatment success was experienced by 80.7% during the first decade, 86.8% during the second decade and 92.1% during the third decade (p=0.003).

Drug resistance was examined in 198 cases, and multi-drug resistant TB was positive in 6.1%. There is no significant difference between ten-year periods regarding multi-drug resistant TB (p=0.666).

## Discussion

In the most recent decade, the number of patients diagnosed with tuberculosis at the NTCD was lower than in the first 10 years but higher than in the second decade. In the 1980s, the incidence of TB in our country was greater compared to the second decade as well as other developing countries. TB incidence has decreased in second decade due to impact of the fight against tuberculosis and socio-economic development. However, the fight against TB has declined in the last decade in Turkey; for example, some dispensaries have been closed by Ministry of Health. Both closure of a Dispensary in Bursa and the increase in population due to internal migration in City may have led to increasing the number of patients in last decade.

Social Security Institution (SSI) patients were registered at TCDs with the transfer of SSI hospitals to the Ministry of Health.^8^ These changes of this study, although parallel the rises and falls throughout all the Turkey, may be related to the patient registration and notification system, the related screening programs, patients’ applications to the TCDs and the registering of SSI patients in the national TB program.

Similar to other studies carried out in Turkey and throughout the world, this study found a higher proportion of males than females among TB patients (throughout Turkey, 58.6%; in the USA, 62%).^13^,^14^,^15^–^18^ Men are more likely than women to participate in social and working life and therefore more often exposed to infection. In addition to male- and female-specific biological and epidemiological differences, the low number of female tuberculosis case notifications can be explained with to the difficulties that woman face in access to health services due to various socioeconomic and cultural factors.^19^–^21^ In this study, the proportion of female patients was significantly higher in the most recent decade compared with the previous ten-year periods. This datum can be explained by an increase of females’ participation in social life and work life and improved access to health services as well as with a real increase in incidence of TB due to worsening economic and living conditions.

The age distribution of tuberculosis patients is also an important indicator in determining the changes in TB’s epidemiology. In developed countries, tuberculosis is most common among the elderly and mostly results from the reactivation of a previous primary infection; however, in developing countries, TB affects all age groups, especially youth and young adults.^22^–^24^ Throughout Turkey, 5.4% of those who received treatment between 2005 and 2011 were in the 0–14 year age group, 58.3% were in the 15–44 year age group, and 11.1% were in the 65 and over age group.^17^ In the USA, 5 % of tuberculosis patients are below the age of 15 years, 40% of them are in the 15 – 44 year age group, and 24 % of them are over 65 year-old.^18^ In our study, although the majority of the patients were in the 15–44 year age group, in line with nationwide studies of Turkey and other studies, the proportion of patients who were younger than 15 years was lower.^14^,^17^,^25^ When ten-year periods were compared, the proportion of patients who were below the age of 15 years was significantly lower in the first decade than in the most recent decade whereas that of the 45–64 year age group and the over 65 age was higher. Although this indicates that the disease moved from the young age group towards an older age group, the change may have resulted from the ageing of the population, the fact that elderly people are more prone to infections and the difficulty of diagnosing tuberculosis infections in childhood in the past.^23^,^24^,^26^

A low level of education is a risk factor for TB at the individual level.^27^ In studies carried out in Van and Isparta in Turkey, the proportions of TB cases with an elementary educational attainment or below are 83.3% and 66.3%, respectively higher than in this study (54.9%).^28^,^29^ The proportion of patients with nine or more years of education increased linearly between ten-year periods for females (p=0.005) and males (p<0.001). This increase may have been related to the increase in the general education level of the patients who visited the NTCD and of the country’s population in general; furthermore, it indicates that tuberculosis does not affect only people with lower educational levels.

The percentages of occupation, business and working status along and the indicators related to the working lives of the patients varied across the studies. In this study, the percentage of unemployed patients was 6.2%, which was lower than in other studies. The percentage of employed patients was 53.0%, which was higher than in other studies.^14^,^19^ In a study carried out in North America, TB was associated with working in non-professional jobs and with unemployment.^27^ In this study, the percentage of those working in jobs that did not require qualification was significantly higher among workers, and the increase in this category in the most recent decade was statistically significant. The sociocultural characteristics of the patients, poor working conditions in jobs that did not require qualification, subcontracted labor, crowded living conditions and screening programs in workplaces are considered important reasons for this increase.

The relapsed cases can be related to insufficient previous treatment, re-exposure to infections, endogenous reactivation of pulmonary or extrapulmonary focus of infection among TB patients and having multidrug-resistant TB. In a study carried out in Brazil, the relapse rate was reported as 5.5% and was associated with high mortality.^30^ This study had a lower percentage of new cases than other studies, but a higher relapse rate.^14^,^29^ The relapse rate decreased in the second decade, tended to increase in the third decade and was significantly higher among patients aged 40 years and older. The high relapse rate may have been due to the high rate of treatment abandonment or happens because the directly observed treatment could not be implemented effectively, or because of inadequate or inappropriate treatment regimens, drug resistance, or advanced age.

The most contagious patients produce living TB bacilli with sputum. The contact time with a patient producing Koch bacilli and the intensity of the bacilli are the risk factors in the development of infection.^31^ The reported history of contact is 8.5%, 13.5% and 38.8% in various studies.^14^,^15^,^29^ The present study found that the history of contact was high in females and children with pulmonary tuberculosis, similar to other studies.^26^,^32^,^33^ In this study, the patients’ contact rate with tuberculosis patients was higher in the last decade than in the first decade and was lower than in the second decade (p=0.006). Among the patients as a whole, TB contact history rate was 19.4%, but diagnosed by contact examination for TB rate was 8% and this rate was higher in the last decade than in the first decade but lower than in the second decade (p=0.030). The contact examinations are important for identifying hidden TB patients who do not visit the dispensary or who wait until late presenter patients and children who are infected as a result of close contact with infectious adult and adolescent TB patients. The high contact rate in the NTCD region paired with a low contact examination rate means that it is necessary to improve contact examinations. In Turkey, the percentage of extrapulmonary TB cases was reported to be 27.0% in 2005, 36.8% in 2011 and 36.0% in 2014.^6^,^17^ In 2011, 27.3% of the male patients and 50.2% of the female patients had extrapulmonary involvement.^17^ In the study by Özkara et al. in 108 dispensaries, the rate of extrapulmonary TB was reported to vary between 14.8% and 28.4% between provinces.^34^ In this study, although the rate of extrapulmonary TB was significantly lower in males, relapse cases and patients with a history of contact in the last decade, it was significantly higher in females^14^,^35^,^36^ and those younger than 15 years^35^ and most frequently showed pleura and lymph node involvement,^30^,^37^ similar to some domestic and foreign studies. In studies carried out in the United States and Brazil, drug resistance was reported to be low in extrapulmonary TB patients.^30^,^36^ Extrapulmonary tuberculosis can be affected by comorbidities that cause immunodeficiency, such as HIV infection; patient characteristics; endocrine factors; immunological and genetic susceptibilities; and differences in endemic tuberculosis strains by population and geographical region, and these factors should be identified.^30^,^35^,^36^ Extrapulmonary tuberculosis had different epidemiological characteristics and risk factors from pulmonary tuberculosis; it can become infectious if it spreads to the lungs, but it is otherwise not infective. Therefore, it is important to determine the risk factors of extrapulmonary tuberculosis for early diagnosis and treatment.

In this study, similar to others, TB cases were most often accompanied by coughing symptoms upon admission in 25.4% to 80% of patients (in Isparta in Turkey 25.4%, in Los Angeles 72.7%, in Samsun in Turkey 80% of patients).^29^,^38^,^39^ In different studies, the other symptoms upon admission were weight loss in 44.5% to 62.9%, anorexia in 11.7% to 59.7%, night sweating in 16% to 54.0% and fever in 52.3%.^29^,^38^,^39^ In the study carried out in Los Angeles, the presence of coughing, fever, weight loss and hemoptysis was associated with health insecurity and a negative tuberculin skin test.^38^ The complaints of malaise (p<0.001), anorexia (p<0.001), weight loss (p<0.001) and coughing (p=0.044) decreased in the most recent decade compared with the previous decades. These findings may indicate that diagnosing TB based on classic symptoms is less possible.

The meta-analysis by Colditz et al. reported that the BCG vaccine decreased the tuberculosis infection risk by an average of 52.0–74.0% and also decreased the deaths from lung tuberculosis, meningitis, miliary and tuberculosis in newborns.^40^ However, the BCG vaccine does not protect from primary infection or reactivation of latent pulmonary tuberculosis, which is the main source for the spread of the bacilli in the community.^41^ In a study carried out in Izmir, the percentage of 0 – 14-year-olds with a BCG scar was determined to be 74.6%.^33^ In the present study, the proportion of patients with a BCG scar was 84.6% among the 0–14 age group and was significantly lower among those aged 40 years and older. The overall percentage of patients with a BCG scar and the percentage of patients aged 40 years and older with a BCG scar have increased at a statistically significant rate in the most recent decade. This increase may also occur due to vaccination of some cases in BCG campaigns across the country that took place between 1953 and 1967.^42^ There was no significant difference between those with a BCG scar and those without a BCG scar regarding pulmonary and extrapulmonary organ involvement. Among those with BCG scar, the mortality rate was significantly lower. Along with the need for further studies that demonstrate the protectiveness of the BCG vaccine and its effect on mortality, these results suggest the need for other, more effective vaccines to prevent latent tuberculosis as well.

The WHO aims to identify at least 70% of smear-positive tuberculosis patients bacteriologically and to treat at least 85% of them successfully. The percentages of treatment success and case identification are the outcomes used to measure the effectiveness of tuberculosis control.^43^,^44^ In this study, among patients with pulmonary TB, the percentages with examined bacteriologically and positive bacteriology were 77.3% and 62.3%, respectively, which are consistent with the values determined in other studies.^34^,^39^

In the study by Özkara et al.,^34^ the treatment success of new pulmonary TB cases with positive bacteriology was 82.5%, which is lower than the value determined in the present study (87.1%). In the present study, while treatment success was significantly higher in the most recent decade than in the previous decades, the percentages of patients who were cured and those who abandoned treatment were significantly lower. “Treatment success” means the total of treatment completion and cure rates. The increase of the treatment success may result from decrease in treatment abandonment along with the increase in treatment completion. The lack of request for bacteriological examination in the end of therapy and the patients who were unable to sputum specimens could be the reason for low cure rates.

A study carried out in Brazil found that being over the age of 40 years, concomitant HIV infection, illiteracy, severe extrapulmonary TB and re-treatment after relapse were risk factors associated with mortality.^30^ In this study, all of the 26 observed deaths occurred in patients aged 40 years and older. There was no significant difference in mortality between the pulmonary and extrapulmonary TB patients. The overall mortality rate was 3.6% in the most recent decade, which was significantly higher than that of the previous decades (p<0.001). The fact that the mortality rates were significantly lower in the past decades may arise from an inability to determine deaths because of high rates of treatment abandonment and low presence of chronic diseases. The mortality rate was high among elderly people (p<0.001), males (p=0.038), patients without a BCG scar (p=0.012), patients with multi-drug resistant TB (p=0.033) and patients with accompanying chronic diseases (p<0.001). All of the deaths were accompanied by at least one chronic illness. The increase in mortality rates among patients treated at the NTCD may have resulted from the growth in the elderly population. This age group has a higher risk of contracting TB, but more frequently presents the masking of the symptoms for comorbidities, a late diagnosis arising from a lack of sufficient care, and a low compliance and the occasional use of drugs because of their side effects. Further studies are needed to determine risk factors and the reasons for the recent increase of mortality to reduce the spread of TB and the deaths.

## Strengths and Limitations of the Study

The fact that the study covers an extensive period of 30 years and the high number of cases reported are the strength of this survey, which analyzes only the data and risk factors that were defined in the records. The TB patients treated at the NTCD were not only residents of the Nilufer district of Bursa but also of some other districts that the dispensary serves. Therefore, the TB prevalence and incidence for the Nilufer district could not be calculated in this study.

## Conclusion

The number of patients treated for tuberculosis in the NTCD was lower in the most recent decade than in the first decade of the survey and higher than in the second decade. In the last ten years, the proportion of females, elderly people and patients with nine years of education or more increased significantly, and of workers among female patients. However, low income countries should further reduce the obstacles related to gender in the diagnosis and treatment of tuberculosis by making structural and social changes, such as increasing women’s socioeconomic circumstances and education levels, reducing discrimination and implementing strategies to remove gender inequalities in health. Unfortunately, the mortality rate also significantly increased probably determined by higher number, pulmonary tuberculosis with positive bacteriology, of elderly people, with accompanying chronic diseases, resistance to antibiotic could also have a role.

It is necessary to increase the efficiency of the control programs and laboratories to detect patients with active pulmonary tuberculosis and efficiently treat them avoiding their contact with risk groups, infants, older and immunodeficient people. These procedures could reduce the spread of infection within the community. The effectiveness of tuberculosis control programs can improve by continuous training of health workers and population, and by functioning of a registration system should to ensure the reliability of patient data. It is necessary to design extensive epidemiological studies that examine the effectiveness of the fight against tuberculosis also in low-income countries, the risk factors and patient mortality and reveal the causal factors. It must not be forgotten that tuberculosis is a social disease that is affected by the inequality in health, and efforts should be made to bring services to patients who cannot reach healthcare services on their own.

## Figures and Tables

**Table 1:** Gender and age distribution of the cases according to ten-year periods

Variable	Ten-years periods					
	1985–1994	1995–2004	2005–2014	Overall		
	N (%)	N (%)	N (%)	N (%)		
**Gender (N=1630)**						
**Female**	168 (26.8)	180 (36.7)	220 (42.9)	568 (34.8)	32.716	p<0.001
Male	459 (73.2)	310 (63.3)	293 (57.1)	1062 (65.2)		
Age Groups (Years)* **(N=1610)**						
**0–14**	35 (5.7)	10 (2.1)	15 (3.0)	60 (3.7)	41.303	p<0.001
15–39	388 (62.9)	263 (54.2)	247 (48.6)	898 (55.8)		
**40–64**	168 (27.2)	166 (34.2)	192 (37.8)	526 (32.7)		
65 +	26 (4.2)	46 (9.5)	54 (10.6)	126 (7.8)		

* There was no data available for 20 cases.

**Table 2:** Socio-demographic characteristics of the cases according to ten years periods.

Gender	Variable	Ten-years periods				χ^2^	P
		1985–1994	1995–2004	2005–2014	Overall		
		N (%)	N (%)	N (%)	N (%)^1^		
**Age Groups (Years)**^3^							
**Female**	0–14	14 (8.5)	7 (3.9)	8 (3.7)	29 (5.2)	21.904	p<0.001
	15–39	114 (69.1)	102 (57.0)	110 (50.5)	326 (58.0)		
	40–64	31 (18.8)	47 (26.3)	74 (33.9)	152 (27.0)		
	65 +	6 (3.6)	23 (12.8)	26 (11.9)	55 (9.8)		
**Male**	0–14	21 (4.7)	3 (1.0)	7 (2.4)	31 (2.9)	23.234	p<0.001
	15–39	274 (60.6)	161 (52.6)	137 (47.2)	572 (54.6)		
	40–64	137 (30.3)	119 (38.9)	118 (40.7)	374 (35.7)		
	65 +	20 (4.4)	23 (7.5)	28 (9.7)	71 (6.8)		
**Marital Status**^4^							
**Female**	Single	39 (26.4)	50 (28.9)	51 (24.5)	140 (26.5)	0.819	p=0.365
	Married	105 (70.9)	101 (58.4)	143 (68.8)	349 (66.0)		
	Divorced, Widowed	4 (2.7)	22 (12.7)	14 (6.7)	40 (7.5)		
**Male**	Single	166 (38.7)	109 (35.5)	96 (34.8)	371 (36.6)	0.829	p=0.362
	Married	252 (58.7)	192 (62.5)	174 (63.0)	618 (61.1)		
	Divorced, Widowed	11 (2.6)	6 (2.0)	6 (2.2)	23 (2.3)		
**Education level**^5^							
**Female**	Illiterate	17 (12.6)	20 (12.4)	13 (7.4)	50 (10.6)	12.966	p<0.001
	Literate	8 (5.9)	6 (3.7)	8 (4.6)	22 (4.7)		
	Primary school graduate	67 (49.6)	65 (40.4)	63 (36.0)	195 (41.4)		
	Secondary school graduate	6 (4.4)	19 (11.8)	17 (9.7)	42 (8.9)		
	High school graduate	31 (23.0)	30 (18.6)	43 (24.6)	104 (22.1)		
	Undergraduate, graduate and postgraduate	6 (4.5)	21 (13.1)	31 (17.7)	58 (12.3)		
**Male**	Illiterate	9 (2.3)	11 (3.7)	2 (0.8)	22 (2.4)	42.611	p<0.001
	Literate	12 (3.0)	7 (2.4)	3 (1.2)	22 (2.3)		
	Primary school graduate	236 (59.3)	136 (45.9)	91 (37.5)	463 (49.4)		
	Secondary school graduate	54 (13.6)	37 (12.5)	41 (16.9)	132 (14.1)		
	High school graduate	69 (17.3)	68 (23.0)	71 (29.2)	208 (22.2)		
	Undergraduate, graduate and postgraduate	18 (4.5)	37 (12.5)	35 (14.4)	90 (9.6)		
**Working status**^6^							
**Female**	Worker	23 (15.5)	27 (15.8)	51 (26.1)	101 (19.6)	6.572	p=0.010
	House wife	110 (74.3)	119 (69.6)	110 (56.4)	339 (66.0)	12.609	p=0.001
	Retired	1 (0.7)	6 (3.5)	15 (7.7)	22 (4.3)	10.337	p<0.001
	Student	12 (8.1)	16 (9.4)	14 (7.2)	42 (8.2)		
	Unemployed	2 (1.4)	3 (1.7)	5 (2.6)	10 (1.9)		
**Male**	Worker	319 (76.9)	216 (72.7)	146 (58.4)	681 (70.8)	23.962	p<0.001
	Retired	28 (6.7)	30 (10.1)	55 (22.0)	113 (11.8)	35.631	p<0.001
	Student	18 (4.3)	20 (6.7)	18 (7.2)	56 (5.8)		
	Unemployed	38 (9.2)	19 (6.4)	25 (10.0)	82 (8.5)		
	Soldier	12 (2.9)	12 (4.1)	6 (2.4)	30 (3.1)		

1 The percentage of the columns.

2 Linear-by-Linear Chi-square test was used.

3 There was no data available for 20 cases.

4 Cases aged 15 years and above were evaluated. There was no data available for 29 cases.

5 Cases aged 15 years and above were evaluated. There was no data available for 162 cases.

6 Cases aged 15 years and above were evaluated. There was no data available for 94 cases.

**Table 3:** Distribution of some variables related to diagnosis and treatment according to ten-year periods.

Variable	Ten-years periods				χ^2^	p
	1985–1994	1995–2004	2005–2014	Overall		
	N (%)	N (%)	N (%)	N (%)		
**Case Definition (n=1538)**						
**New case**	479 (85.1)	421 (87.7)	434 (87.7)	1334 (86.7)	15.218	p=0.037(1)
Relapse	65 (11.5)	39 (8.1)	45 (9.1)	149 (9.7)		
Treatment after default	4 (0.7)	2 (0.4)	0 (0.0)	6 (0.4)		
Chronic case	6 (1.1)	1 (0.2)	1 (0.2)	8 (0.5)		
Transfer in	9 (1.6)	17 (3.6)	15 (3.0)	41 (2.7)		
**Reason for admission/examination (n=1480)**						
**İndividual**	472 (87.2)	400 (84.2)	393 (84.7)	1265 (85.5)	7.017	p=0.030
Contact	35 (6.5)	51 (10.7)	33 (7.1)	119 (8.0)		
Organized	5 (0.9)	1 (0.2)	12 (2.6)	18 (1.2)		
For report	29 (5.4)	23 (4.9)	26 (5.6)	78 (5.3)		
**Symptoms (n=1328)**						
Malaise	178 (32.9)	13 (28.2)	90 (21.3)	391 (27.9)	15.766	p<0.001 (2)
Anorexia	159 (29.4)	73 (16.7)	47 (11.1)	279 (19.9)	51.392	p<0.001 (2)
Weight loss	216 (39.9)	98 (22.5)	86 (20.3)	400 (28.6)	47.581	p<0.001 (2)
Cough	411 (76.0)	300 (68.8)	298 (70.4)	1009 (72.1)	4.067	p=0.044 (2)
Back pain	16 (2.6)	14 (2.9.9	28 (5.5)	58 (3.6.9	6.632	p=0.010 (2)
**Previously tuberculosis (n=1527)**						
**No**	492 (86.9)	430 (91.3)	444 (90.6)	1366 (89.5)	4.023	p=0.05 (2)
Yes	74 (13.1)	41 (8.7)	46 (9.4)	161 (10.5)		
**Contact with TB patient (n=1320)**						
**Yes, domestic**	63 (12.9)	95 (21.2)	70 (18.3)	228 (17.3)	14.596	p=0.006
Yes, non-domestic	13 (2.7)	11 (2.5)	4 (1.0)	28 (2.1)		
No	413 (84.5)	342 (76.3)	309 (80.7)	1064 (80.6)		
**BCG scar (n=1115)**						
**Yes (1 and above scar)**	308 (72.8)	246 (73.4)	308 (86.3)	862 (77.3)	24.101	p<0.001 (2)
No	115 (27.2)	89 (26.6)	49 (13.7)	253 (22.7)		
**Involvement Site**(3) **(n=1604)**						
**Pulmonary**	506 (82.0)	345 (71.4)	337 (66.9)	1188 (74.1)	34.016	p<0.001 (2)
Extrapulmonary	111 (18.0)	138 (28.6)	167 (33.1)	416 (23.9)		
**Treatment Outcome (n=1618)**						
Treatment success (4)	524 (84.1)	428 (87.5)	462 (91.3)	1414 (87,4)	13.112	p<0.001 (2)
[Treatment completion]	[301 (48.3)]	[352 (72.0)]	[407 (80.4)]	[1060(65.5)]	140.489	p<0.001 (2)
[Cure]	[223 (35.8)]	[76 (15.5)]	[55 (10.9)]	[354 (21.9)]	105.814	p<0.001 (2)
Treatment abandonment (5)	79 (12.7)	28 (5.7)	11 (2,2)	118 (7.3)	30.226	p<0.001 (2)
Treatment failure	1 (0.1)	4 (0.8)	1 (0.1)	6 (0.4)		
Death	1 (0.2)	7 (1.5)	18 (3.6)	26 (1,6)	20.107	p<0.001 (1)
Transfer out	18 (2.9)	22 (4.5)	14 (2.8)	54 (3,3)		

(1) Fisher’s exact test was used.

(2) Linear-by-Linear Chi-square test was used.

(3) Miliary TB and pulmonary + extrapulmonary TB are located within the pulmonary TB.

(4) Treatment success= treatment completion + cure

(5) Treatment outcome unknown of TB cases are included in this group.

**Table 4:** Distribution of gender, age group, and involvement site among cases according to contact history, BCG scar presence and relapse.

	G scar presence								
	N (%)*	χ^2^	p	N (%)*	χ^2^	p	N (%)*	χ^2^	p
**Gender**									
Female	112 (24.0)	9.922	0.002	296 (77.9)	0.113	0.737	47 (8.8)	0.795	0.373
Male	144 (16.9)			566 (77.0)			102 (10.2)		
**Age Groups**									
0–14	16 (29.6)	1.428	<0.001	33 (84.6)	108.083	<0.001	1 (1.7)	30.454	<0.001
15–39	168 (22.9)			558 (86.1)			58 (6.9)		
40–64	60 (14.4)			231 (68.1)			77 (15.4)		
65 +	10 (10.1)			30 (39.5)			12 (10.3)		
**Involvement site**									
Pulmonary	202 (20.8)	7.448	0.006	627 (75.9)	2.963	0.085	132 (11.8)	21.088	<0.001
Extrapulmonary	46 (14.0)			221 (81.0)			15 (3.8)		

* The percentage of row.

**Table 5:** Distribution of gender, age group and treatment success among cases according to involvement site.

Variable	Pulmonary TB	Extrapulmonary TB	Overall	χ^2^	p
	N (%)*	N (%)*	N (%)*		
**Gender**					
**Female**	337 (28.4)	225 (54.1)	562 (35.0)	89.544	p<0.001
Male	851 (71.6)	191 (45.9)	1042 (65.0)		
**Age groups (years)**					
**0–14**	29 (2.5)	31 (7.6)	60 (3.8)	21.822	p<0.001
15–39	673 (57.2)	211 (51.6)	884 (55.7)		
40–64	392 (33.3)	125 (30.5)	517 (32.6)		
65 +	83 (7.0)	42 (10.3)	125 (7.9)		
**Treatment success**					
**Yes**	1019 (85.9)	379 (92.2)	1398 (87.5)	11.088	p=0.001
No	167 (14.1)	32 (7.8)	199 (12.5)		

* The percentage of column.

**Table 6:** Distribution of the bacteriology of pulmonary TB cases according to ten-year periods.

Variables	Periods of ten-years				χ^2^	p
	1985–1994	1995–2004	2005–2014	Overall		
	N (%) 2	N (%) 2	N (%) 2	N (%) 2		
**Bacteriology**^1^ **(n=1138)**						
**Positive**	182 (37.4)	151 (45.1)	215 (68.0)	548 (48.1)	78.040	p<0.001
Negative	181 (37.2)	93 (27.7)	58 (18.4)	332 (29.2)		
Could not be made	124 (25.4)	91 (27.2)	43 (13.6)	258 (22.7)		

1 Indicates the culture and/or spread examinations. There was no data available for 50 cases.

2 The percentage of the columns.

## References

[1] World Health Organization WHO tuberculosis.

[2] World Health Organization Global Tuberculosis Report 2015.

[3] World Medical Association Physicians advised to treat tuberculosis as a disease of poverty.

[4] European Centre for Disease Prevention and Control/WHO Regional Office for Europe (2016). Tuberculosis surveillance and monitoring in Europe 2016.

[5] Vidinel I, Bilgic H, Karadag M (2010). A historical view to tuberculosis in Turkey. Toraks Kitaplari Tüberküloz.

[6] World Health Organization WHO tuberculosis.

[7] Özkan S, Bilgic H, Karadag M (2010). Basic Institution of fight against tuberculosis in our country: Tuberculosis Dispensary. Toraks Kitaplari Tüberküloz.

[8] Özkara S, Bilgic H, Karadag M (2010). Tuberculosis Epidemiology in Turkey. Toraks Kitaplari Tüberküloz.

[9] Özdemir T, Türkkani MH, Yilmaz Aydin L, Balci Ç, Danacibas R, Bilgin A (2014). Provincial assessment in the scope of the tuberculosis control program. Tuberk Toraks.

[10] Akdag R, T. R. Ministry of Health (2011). Tuberculosis Diagnosis and Treatment Guidelines 2011.

[11] Turkish Republic Official Newspaper.

[b12-mjhid-8-1-e2016059] 12Turkish Rebuplic Ministry of Health Department of Tuberculosis 16.09.2003 tarih ve 7546 sayili yazisi. (in Turkish).

[13] Koçakoglu S, Simsek Z, Ceylan E (2009). Epidemiologic characteristics of the tuberculosis cases followed up at Sanliurfa central tuberculosis control dispensary between 2001 and 2006 years. Turkish Thoracic Journal.

[14] Orman A, Ünlü M, Cirit M (2002). Assessment of 627 tuberculosis cases observed in Afyon tuberculosis dispensary between 1990 and 2000. Respiratory Diseases.

[15] Martinez AN, Rhee JT, Small PM, Behr MA (2000). Sex differences in the epidemiology of tuberculosis in San Francisco. Int J Tuberc Lung Dis.

[16] Çiftçi F, Bozkanat E, Ilvan A, Kartaloglu Z, Sezer O, Çaliskan T, Kaya H (2006). The year of 2003 treatment results of soldier patients with tuberculosis in a military hospital which has feature of the referance. Turk Thorac J.

[17] Özkan S, Musaonbasioglu S, Turkish Republic Ministry of Health, Public Health Institution of Turkey Department of Tuberculosis (2014). Tuberculosis struggle in Turkey 2013 Report.

[18] CDC (2015). Reported Tuberculosis in the United States, 2014.

[19] Borgdorff MW, Nagelkerke NJD, Dye C, Nunn P (2000). Gender and tuberculosis: a comparison of prevalence surveys with notification data to explore sex differences in case detection. Int J Tuberc Lung Dis.

[20] Neyrolles O, Murci LO (2009). Sexual inequality in tuberculosis. PLoS Medicine.

[21] Hudelson P (1996). Gender differentials in tuberculosis: the role of socioeconomic and cultural factors. Tuber Lung Dis.

[22] Tatar D, Keskin Ö, Özacar R, Halilçolar H (2002). Similarity and differences of tuberculosis in the young and the elderly patients. Tuberculosis and Thorax.

[23] Öztop A, Ünsal I, Günay T, Özgü A, Çakmak R, Uçku R (2006). The epidemiological characteristics of tuberculosis patients registered in Kahramanlar dispensary between 1999 and 2003. Respiratory Diseases.

[24] Kiliçaslan Z, Bilgic H, Karadag M (2010). Epidemiology of Tuberculosis and Tuberculosis in the World. Toraks Kitaplari Tüberküloz.

[25] Kiter G, Coskunol I, Alptekin S (2000). Evaluation of tuberculosis patients enrolled to Esrefpasa tuberculosis dispensary between january 1997 to june 1998. Tuberculosis and Thorax.

[26] Sen V, Uluca Ü, Yilmaz S, Selimoglu Sen H, Tuncel T, Günes A, Karabel M, Gürkan MF (2014). The evaluation of the clinical and laboratory characteristics of children with pulmonary tuberculosi. Dicle Medical Journal.

[27] Young BN, Rendon A, Rosas A, Baker J, Healy M, Gross JM, Long J, Burgos M, Hunley KL (2014). The effects of socioeconomic status, clinical factors, and genetic ancestry on pulmonary tuberculosis disease in northeastern mexico. PLOS ONE.

[28] Özbay B, Sezgi C, Altinöz O, Sertogullarindan B, Tokgöz N (2008). Evaluation of tuberculosis cases detected in our region between 1999 and 2003. Tuberculosis and Thorax.

[29] Zengin E, Kisioglu AN, Sönmez Y (2009). The characteristics of tuberculosis cases which registered in Isparta central district tuberculosis dispensary and the sufficiency situation of dispensary registries: 2000 to 2007 years. SDÜ Tip Fakültesi Dergisi.

[30] Gomes NMDF, Bastos MCDM, Marins RM (2015). Differences between risk factors associated with tuberculosis treatment abandonment and mortality pulmonary medicine.

[31] Öztürk R (2003). Natural Resistance in Tuberculosis and Risk Factors, Tuberculosis Semposioum in the 21th Century and 2nd Course of Tuberculosis Diagnosis Methods in the Laboratory Samsun. http://www.klimik.org.tr/wp-content/uploads/2012/02/982011124221-Recep_Ozturk.pdf.

[32] Aksel N, Mertoglu A, Dogan H (2008). Comparison of the male and female cases with pulmonary tuberculosis. Izmir Gögüs Hastanesi Dergisi.

[33] Tatar D, Alptekin S, Coskunol I, Aydin M, Arslangiray S (2002). The retrospective analysis of tuberculosis in children followed up at Izmir Esrefpasa dispensary between 1995–2000. respiratory diseases.

[34] Özkara S, Kiliçaslan Z, Öztürk F, Seymenoglu S, Erdogan AR, Tellioglu C, Kosan AA, Kaya B, Koçoglu F, Kibaroglu E (2002). Tuberculosis in Turkey with regional data. Turkish Thorasic Journal.

[35] Forssbohm M, Zwahlen M, Loddenkemper R, Rieder HL (2008). Demographic characteristics of patients with extrapulmonary tuberculosis in Germany.

[36] Peto HM, Pratt RH, Harrington TA, LoBue PA, Armstrong LR (2009). Epidemiology of extrapulmonary tuberculosis in the United States, 1993–2006. Clin Infect Dis.

[37] Kolsuz M, Ersoy S, Demircan N, Metintas M, Erginel S, Alatas F, Uçgun I (2003). The evaluation of pulmonary tuberculosis patients enrolled to Eskisehir Deliklitas tuberculosis control dispensary. Tuberculosis and Thorax.

[38] Miller LG, Asch SM, Emily IY, Knowles L, Gelberg L, Davidson P (2000). A population-based survey of tuberculosis symptoms: how atypical are atypical presentations?. Clin Infect Dis.

[39] Kayhan S (2012). Demographic and clinical characteristics of tuberculosis: A report of 2404 cases at a referral hospital. Afr J Microbiol Res.

[40] Coldwitz GA, Berkey CS, Mosteller F, Brewer TF, Wilson ME, Burdick E, Fineberg HV (1995). The efficacy of bacillus Calmette-Guerin vaccination of newborns and infants in the prevention of tuberculosis: meta-analyses of the published literature. Pediatrics.

[41] World Health Organization Geneva (2004). WHO car Vaccine [online]. Weekly epidemiological record.

[42] Kiliçaslan Z, Koçoglu F, Bilgic H, Karadag M (2010). The Role of the Tuberculosis Society Fight against TB in Turkey. Toraks Kitaplari Tüberküloz.

[43] World Health Organization Stop TB Partnership and World Health Organization. The Global Plan to Stop TB, 2006–2015: actions for life towards a world free of tuberculosis.

[44] Jordan TS, Davies PD (2010). Clinical tuberculosis and treatment outcomes. Int J Tuberc Lung Dis.

